# Towards autonomous robotic THz-based in vivo skin sensing: the PicoBot

**DOI:** 10.1038/s41598-025-88718-6

**Published:** 2025-02-07

**Authors:** Anubhav Dogra, Dominic Jones, Arturo Ignacio Hernandez Serrano, Shruti Chakraborty, Jacob Joshua Young, Benjamin George Page, Joseph Hardwicke, Pietro Valdastri, Emma Pickwell-MacPherson

**Affiliations:** 1 Department of Physics, University of Warwick, Coventry, UK; 2 STORM Lab, University of Leeds, Leeds, UK; 3 Warwick Medical School, University of Warwick, Coventry, UK; 4 Institute of Applied and Translational Technologies in Surgery, University Hospitals Coventry and Warwickshire NHS Trust, Coventry, UK; 5 Present Address: CEA LIST, Université Paris Saclay, Palaiseu, France

**Keywords:** Imaging techniques, Terahertz optics, Skin cancer

## Abstract

Terahertz (THz) light has the unique properties of being very sensitive to water, non-ionizing, and having sub-millimeter depth resolution, making it suitable for medical imaging. Skin conditions including eczema, psoriasis and skin cancer affect a high percentage of the population and we have been developing a THz probe to help with their diagnosis, treatment and management. Our in vivo studies have been using a handheld THz probe, but this has been prone to positional errors through sensorimotor perturbations and tremors, giving spatially imprecise measurements and significant variations in contact pressure. As the operator tires through extended device use, these errors are further exacerbated. A robotic system is therefore needed to tune the critical parameters and achieve accurate and repeatable measurements of skin. This paper proposes an autonomous robotic THz acquisition system, the PicoBot, designed for non-invasive diagnosis of healthy and diseased skin conditions, based on hydration levels in the skin. The PicoBot can 3D scan and segment out the region of interest on the skin’s surface, precisely position (± 0.5/1 mm/degrees) the probe normal to the surface, and apply a desired amount of force (± 0.1N) to maintain firm contact for the required 60 s during THz data acquisition. The robotic automation improves the stability of the acquired THz signals, reducing the standard deviation of amplitude fluctuations by over a factor of four at 1 THz compared to hand-held mode. We show THz results for skin measurements of volunteers with healthy and dry skin conditions on various parts of the body such as the volar forearm, forehead, cheeks, and hands. The tests conducted validate the preclinical feasibility of the concept along with the robustness and advantages of using the PicoBot, compared to a manual measurement setup.

## Introduction

Basal Cell Carcinoma (BCC) is the most prevalent form of skin cancer and is one of the UK’s top 5 common cancers with around 180,000 new cases annually^1^. Skin cancer poses diagnostic challenges as it relies on visual inspection and biopsy by expert physicians. Precise pre-surgical imaging able to accurately determine the excision margins is needed to speed up the surgery, avoid re-emergence and reduce costs. Machine Learning and Artificial Intelligence image analysis^2^ have emerged to segment/categorize visible skin lesions based on white-light image data; however, determining the extent of tumor margins still demands surgical expertise. Terahertz (THz) light shows promise as a non-invasive method to extract tumor margins both on and below the skin’s surface^3^. THz light, with frequencies from 100 GHz to 30 THz (wavelength, $3\, {\text{mm}}$ to $10\, \upmu {\text{m}}$)^4^ and photon energy in the order of $\text{meV}$ is far safer compared to X-rays, which have photon energy in the order of $\text{KeV}$ (a million times higher). Leveraging its water absorption characteristics^5,6^, non-ionizing nature and sub-millimeter resolution^7^, THz light is suitable for medical applications like tumor screening and health monitoring^8,9^, along with various other fields, such as non-destructive testing, paint thickness evaluations, weather monitoring, and homeland security.

The critical requirements for accurate and repeatable THz measurements include perfect alignment of the incident THz light normal to the surface of the sample, micro to millimeter depth resolution window based upon the focal depth of the THz light, and size of the sample to be measured. This is not so straightforward when the sample is an area of skin on a living (in vivo) human as breathing and other movements will affect the positioning. THz-Time Domain Spectroscopy (TDS) systems such as the Menlo TeraSmart system used in this work are available commercially and provide fast and reliable THz scanning. These systems are set up in reflection or transmission geometry depending upon the absorptivity of the sample to be measured. For the thick and highly absorbing samples, such as the skin surface, a reflection setup is used. THz-TDS measures a discrete single point on the surface, which may be raster scanned to produce 2D surface images. This measurement system is typically bulky and immovable, requiring test samples to be manually placed on the device. Previously, we designed a portable handheld probe^10^ to provide in vivo THz-TDS scans on different regions of the body^11^. However, a handheld device, contextualized in a contact or non-contact measurement system, is prone to positional errors through sensorimotor perturbations and tremors, giving spatially imprecise measurements and (in the case of contact scanning) significant variations in contact pressure. As the operator tires through extended device use, these errors are further exacerbated. When considering skin scanning, movement of the patients through respiratory motion or involuntary muscle contractions can compound these errors. The major challenges associated with the requirements of an in vivo THz sensing system are as follows.

1. Segmentation of the region of interest (ROI) on the areas where the dry or diseased skin condition is found (even on the healthy skin regions),
2. Orientation and positioning of the THz probe perpendicular to the ROI at a specified contact pressure.
3. Safe Interaction of the robot with human participants and keeping the THz probe in contact with the constant force on the skin surface for at least 60 s while measuring the reflected THz signal.
4. Compensation for any motion due to breathing.

Recent technical developments have focused on generating THz measurements of the surface of 3D objects with arbitrary shapes, and mounting THz optical architectures on robot end-effectors to provide precise and repeatable measurements. The applications are presented chiefly for industrial environments such as non-destructive testing of PVC pipes^12^, free-form imaging or paint thickness measurements of arbitrarily shaped samples^13,14^, holographic reconstruction of non-metallic samples with unknown structures^15^, and even scanning the ex vivo samples of dummy human remains^16^. A collaborative robot has also been developed with a hand-guiding system for THz raster scanning of a sample placed in 2D space^17^. In all these cases, the measurements are taken on a static sample; the samples are lying on the measurement platform and are still (and non-living). When translated to medical scenarios, however, the critical challenge is to measure in vivo (without anaesthetic); the measurement surface of the skin has an arbitrary curvature and is prone to movement, reducing repeatability and reliability in the measurements.

The medical robotics field has gained significant momentum in the last decade, with applications spanning most aspects of the medical field, driven by the need for higher-quality treatment alongside advancements in robotic and imaging technologies^18^. Robot-assisted medical procedures benefit patient outcomes and provide an ergonomic improvement for practicing clinicians^19,20^, potentially reducing the prevalence of repetitive strain injuries in the hand, wrist, neck, or back of the operator caused by prolonged handling of tools. In robot-assisted medical imaging, the robotic components are designed, and the control is tuned to a single medical task. Of all imaging modalities, ultrasound (US) is the most explored^21,22^, using robots to perform extracorporeal contact scans of the body. Most systems utilize a similar technical setup consisting of a robotic manipulator with end-effector mounted imaging, using combined white-light and depth camera (RGB-D sensor) and force/torque sensing. A simplified process for performing a scan would be planning a probe trajectory across the skin surface and manipulating the probe to follow this path while maintaining adequate coupling contact. In some cases, the planning aspect is handled by manually placing the probe over the target areas^23^ or using pre-interventional MRI or CT scan images to pre-plan an optimal trajectory before registering with the body surface using RGB-D feedback^24–26^. The most versatile method is directly identifying and segmenting the region of interest from the RBG-D sensor, using factors such as skin color^27,28^. Once segmented, the system plans a full-coverage raster path across the area to ensure all regions are adequately imaged. However, these methods suffer somewhat when deployed in an unstructured real-world environment; different body parts, skin color, skin occlusion by clothing, and incorrect placement of the RGB-D lead to inconsistencies, requiring human intervention to improve the performance. During the scanning process, information from the force/torque (F/T) sensor, either attached at the tip of the manipulator or computed through joint torque sensors, is primarily used to maintain good contact between the probe and the skin^29–32^. Additionally, F/T information is used for moving and rotating the probe to maintain perpendicularity with the skin based on soft tissue deformation^31^. More recent methods seek to use the real-time probe data as further sensory feedback, allowing real-time planning of the probe trajectory^33^ or optimization to focus sensing areas of interest previously observed in the scan^34^.

**Fig. 1:**
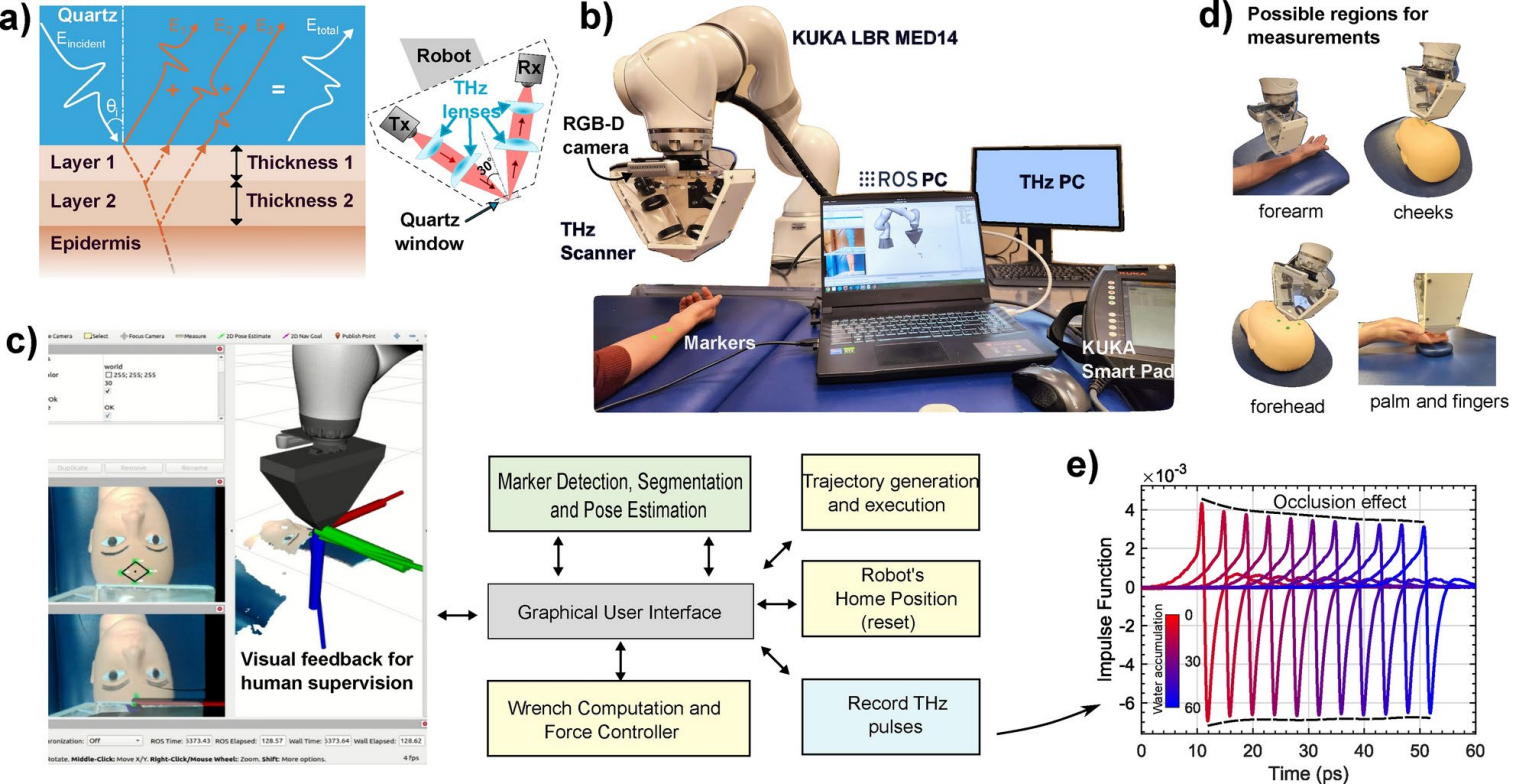
PicoBot System: a complete description of robotic setup with different hardware and software modules. (**a**) Diagram of the probe architecture highlighting the layers that the THz pulse travels through before and after reflection from the skin (**b**) Photograph of the robotic setup including a robotic manipulator, robot and THz controllers, and an RGB-D camera (**c**) Software architecture and visualization of the ROI segmentation and robot motion planning, (**d**) Possible areas of measurements on a human body (**e**) Resulting processed THz pulses (impulse functions) from a single measurement.

The aforementioned challenges led to the main contribution of this work: developing an autonomous robotic patient scanning system, named the PicoBot. The PicoBot can perform $\text{in vivo}$ scans by segmenting out an ROI on the skin surface, precisely positioning the probe, and applying a desired amount of force to maintain firm controlled contact throughout the scan time, as shown in Fig. 1. Our PicoBot can scan various regions of the body, measuring hydration levels of different skin types such as healthy, dry, or diseased skin, which is fundamental to diagnosing skin cancers. Throughout the scanning process, the THz probe makes contact with the skin through a transparent quartz window, flattening the skin and maintaining alignment within the optical setup.

THz light can penetrate the topmost layer of skin, the stratum corneum (SC), into the remaining epidermis below. The reflected THz signal carries information about the structure and hydration of the skin. There is typically a water concentration gradient across the SC which can be modeled using stratified medium theory^10^ to deduce the water concentration profile. When the quartz window covers and presses against the skin, it blocks water vapor exchange, which we refer to here on as “occlusion”, leading to increased surface hydration. The increase in hydration causes the reflected THz signal to be reduced, which in turn reduces the THz impulse function. This response is also affected by the contact pressure. We focus our THz analysis in this paper on how consistently we are able to measure the THz data. Further processing steps to calculate, for example, the skin hydration are given in our previous study^10^. The frequency domain response function, IF, is calculated by dividing the signal reflected off the skin by the signal reflected off the reference window. The time domain impulse function is calculated by multiplying IF by a filter function and then taking the inverse Fourier transform, this enables any fluctuations in the incident THz signal to be accounted for and is explained in more detail in^10^. We refer to the time domain impulse function as the processed THz pulses, as shown in Fig. 1e. IF is a complex variable (with amplitude and phase) and is used to highlight the stability of the PicoBot in Fig. 5c and d. Figure 1e shows how the THz pulse peak-to-peak (P2P) amplitude decreases due to occlusion during the 60 s measurement. The decay rate of the occlusion curve is significant: a faster decay indicates drier skin, while a slow or stable curve suggests well-hydrated skin. The direct comparison of the occlusion curves between the two areas indicates a difference in hydration levels for each area.

The PicoBot is designed for operator interaction through our custom made Graphical User Interface (GUI), Fig. 1c, allowing the user to initialize/interrupt the ROI segmentation, pose estimation, Cartesian motion plans, force controller, wrench estimation using internal torque sensors, and dynamically reconfigure the parameters of impedance and the desired force. It also has a visual feedback system for cross-verification before making contact with the sample—the operator can approve each step after visual inspection.

Our vision is to upgrade this to a fully autonomous system that can perform full body scans without requiring highly specialized personnel (e.g., a dermatologist). This might include autonomous scanning and spotting cancerous skin regions, performing imaging over the entire ROI, non-contact scanning of the ROI, and 3D imaging of the tumor margins. The main objective of this work is to increase the overall precision, reliability and repeatability of the system compared to the manual system and reduce the operator load. Inducing automation for tasks demands quantitative analysis comparing robot vs manual solutions. To have a sense of this, the quantitative analysis concerning the repeatability and reliability of this system is provided in the results section to showcase how the proposed robotic system outperforms the hand-held system.

## Results

In this section, we present the design and the workflow of the PicoBot system followed by the quantitative analysis of the marker-based segmentation and localization, force controller, threshold force, and effect of forces on the THz measurement of the skin. The stability/reliability and repeatability tests on a mannequin as well as on human volunteers are conducted and a comparison of the robotic system with the measurements of the hand-held system is presented. This is followed by showcasing scanning procedures on four different areas of the human body such as the volar forearm, cheeks, forehead, and fingers to highlight the potential of the PicoBot, see Fig. 1d. The experimental protocol is given for every THz measurement routine to be followed. The interaction of THz light with different skin locations on the body, and different skin conditions is explained and compared to healthy skin measurements for the understanding and clarity of the acquired data.

### System description

The complete system, as shown in Fig. 1b, consists of a 7-DoF medical grade manipulator: KUKA LBR Med14, an RGB-D camera: RealSense D435i mounted on the end-effector of the manipulator, and a custom built THz probe. As shown in Fig. 1a, the THz probe works in reflection geometry with an incident angle of ${30}^{\circ }$ and is attached to the manipulator’s end-effector. The tip of the probe has a transparent quartz window of diameter $10\, \text{mm}$. The tip is designed in a way to be at the focal point of the two THz lenses closest to the skin. The quartz window also helps to flatten the skin under examination improving the efficiency of the reflected THz light. The proposed THz system sends broadband electromagnetic pulses $\left({\text{E}}_{\text{incident}}\right)$ onto the skin. These pulses experience multiple reflections from the interfaces of the different layers in the skin $\left({\text{E}}_{1}, {\text{E}}_{2},{\text{ E}}_{3}\right)$ and are collected as the total electric fields detected by the THz receiver $\left({\text{E}}_{\text{total}}\right).$ The total electric field carries information about the structure of the skin as well as the hydration values in the layers interacting with the THz pulse. The system also includes a 12th gen Intel i7-12700H computer with a clock speed of 2.70 GHz on which the Robot Operating System (ROS) is running and executing all the necessary nodes responsible for manipulators motion planning and execution, Cartesian impedance behavior, tool-tip force control, vision-based target detection, and localization. To control each node, a custom Graphical User Interface is designed for easy human–robot interfacing, through which a user can start/stop a group of nodes during measurements if necessary, see Fig. 1c.

### Measurement protocol

Before conducting experiments, a consent form was completed by all participants, followed by an approved short survey about their recent activities which may affect skin hydration. Acclimatization of the participants with the indoor environment was done to aid meaningful comparisons. Patients were requested to lay on the adjustable bed to improve target region localization and the tooltip manipulability. This allowed the system to be in its dexterous workspace and avoid large motion trajectories from the start pose to the scanning pose. A Graphical User Interface is developed for easy interaction of the operator with the PicoBot, which can be used to initialize the robot startup. Marking of the target area to be measured or scanned is performed and then the measurement region of interest (ROI) is segmented from the RGB-D images using marker-based techniques proposed in this paper. The robot is moved to a safe distance of $10 mm$ above the skin surface before the force controller loop is activated. Once skin-probe contact is achieved, the force controller maintains the desired force for 60 s while the THz pulses are recorded. The acquired THz pulses are post-processed for further evaluation; occlusion curves, refractive indices, hydration levels, and skin thickness are calculated and are compared to the healthy skin region, if necessary^10^.

### Quantitative analysis

**Fig. 2:**
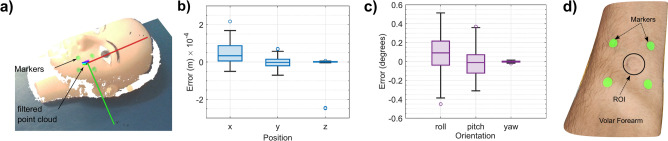
Repeatability analysis for the marker-based pose estimation. (**a**) Pose estimation based on the marker-based strategy on a face mannequin. (**b**) Box chart for the errors in estimating the position of the target pose from repeated measurements when the robot is still at the home position. (**c**) Box chart for the errors in estimating the orientation represented in Euler angles of the target pose from repeated measurements. (**d**) Snapshot of the volar forearm with four markers and ROI where the probe was made to contact during the repeated human trials.

For the Segmentation and localization of the ROI on any skin type (healthy or diseased), we use a marker-based strategy. Four green markers are used to crop the ROI, as shown in Fig. 2a and d, using an RGB-D camera, and the resulting point cloud data is processed for estimating a 3D pose for the robot, as detailed in the methods section. To quantify the precision of marker-based estimation of the pose (target), five repeated estimations of the pose were done on a marked ROI on a mannequin, see Fig. 2a. The target pose is estimated from the set home/start position of the robot in the setup for all five repeated tests. This is important because we would want the robot to always reach the desired/target location with the desired orientation for accurate THz measurements. The target pose is estimated from the set home position of the robot in the setup for all five repeated tests. As shown in Fig. 2b and c, the max error for the position and orientation of the estimated pose is approximately less than 0.5 mm and 1 degree, respectively. A snapshot of the volar forearm post-repeated measurements is shown in Fig. 2d to highlight the repeatability of the position of the probe during human trials.

**Fig. 3:**
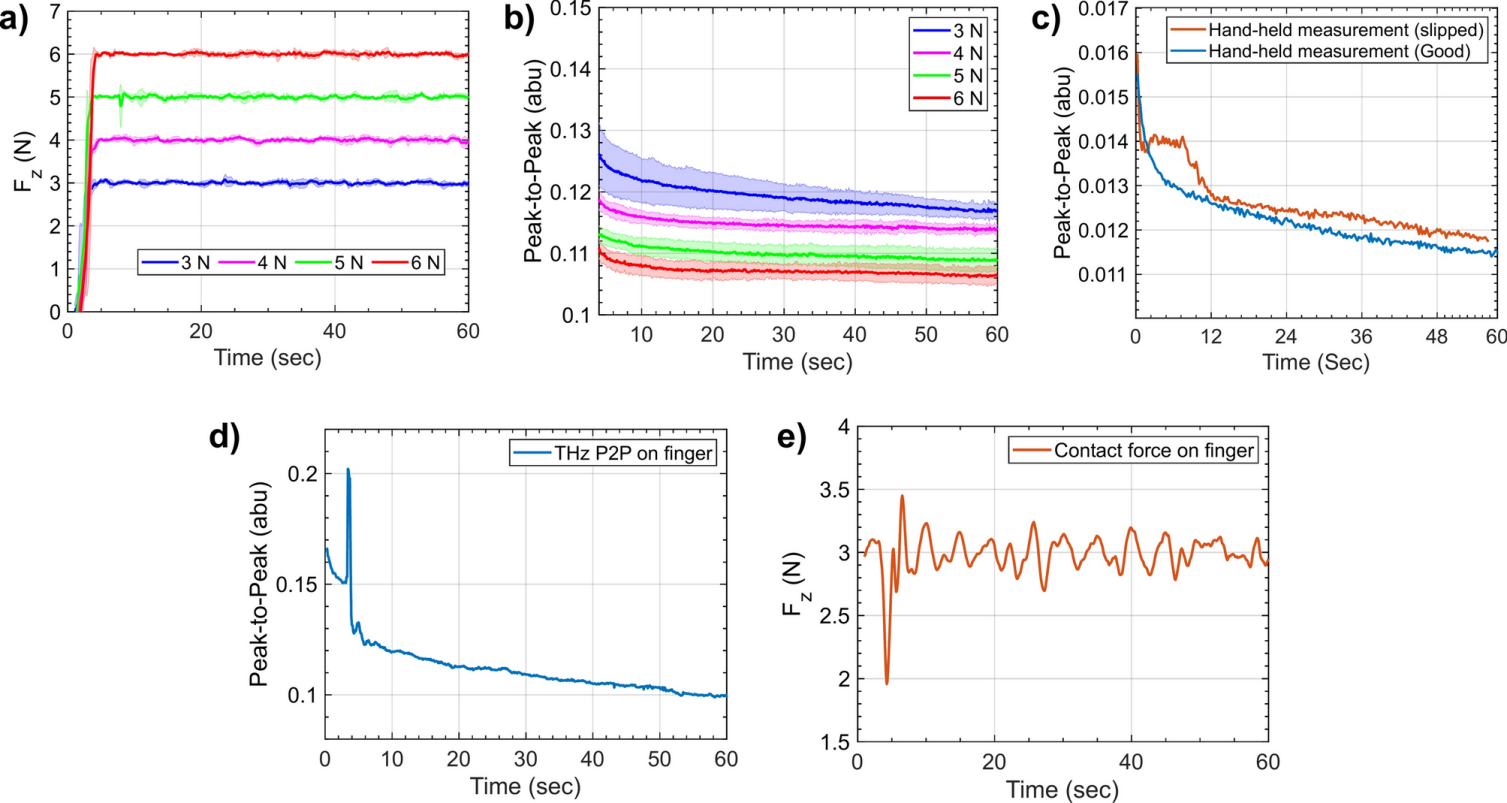
Repeatability analysis for the measurement scans using the PicoBot with different forces and a comparison with the handheld probe. (**a**) Shaded error bars are shown for the forces applied when acquiring the results presented in (**b**), showcasing the stability of the force applied. (**b**) The peak-to-peak of the occlusion curves during a 60 s measurement of the volar-forearm of a subject with increasing applied forces by the robot. The measurements at each of the four forces were performed five times. The shaded error bars represent the standard deviations from the mean value for each set of five measurements (**c**) A measurement of a volar forearm using the handheld probe in a good measurement (blue line) and a measurement where the probe operator slipped (red line). (**d**) Inconsistent THz measurement when the finger was moved deliberately. (**e**) Corresponding force feedback with respect to (**d**).

A set-point-based force controller is designed using the Cartesian-Impedance behaviour of the PicoBot system. The end-effector wrench values are computed via internal joint torque sensors present in the manipulator, and using the kinematic information of the manipulator configuration. The consistency of the force controller is shown in Fig. 3a, for which the THz measurements were performed by the PicoBot on the volar forearm of a volunteer for 5 times and with 4 different forces of 3, 4, 5, and 6N, counting to a total of 20 measurements. The measured force over the skin has shown repeatability with a variance of 1.5–3% for each measurement. To check the repeatability of the PicoBot when applying force on the human skin surface is better to look at the resulting THz measurements. Previous studies have shown that the applied pressure on the skin surface affects the THz response—a higher force results in a lower peak-to-peak amplitude, in turn affecting the occlusion curve of the skin^35^*.* Our PicoBot measurements are consistent with this and we have carefully investigated the threshold force to be used for the measurements.

Figure 3b indicates the clear distinction between the occlusion curves when the robot applied different loads over the skin on the volar forearm. For the measurements shown in Fig. 3b, the variance for each of the measurements ranges from 0.5 to 2%. The difference between levels of peak-to-peak curves indicates the difference in the level of hydration in the Stratum Corneum (SC) of the skin, or the skin thickness of the subject^35^. From the participants’ experiences, and analysing processed data, it was decided to use the force value between 3–4N, in the case of the volar forearm, for the contact measurements. Figure 3c showcases what happens to the P2P signal if the operator slips during the measurement while measuring in the hand-held mode. Furthermore, there is no other way to track this slip, and the operator does not even feel the slight slipping of the device along the surface of the skin. In contrast, the PicoBot never slips, and so the only issue along these lines is if the subject moves suddenly such that robot cannot track the measurement location. Figure 3d shows a measurement of a volunteer who deliberately moved at about 5 s into the measurement by the PicoBot. This manifests as inconsistency in the THz P2P trend and is also verified by the force signal recorded—a spike in the force is seen at 5 s in Fig. 3e. Thus, the force recordings can be used to clarify the origin of any inconsistencies in the THz measurements if found. Indeed, we use our real-time tracking to inform us about the progress of measurements and if there are any large variations in the force, we can re-take the data.

### Robotic vs. manual data acquisition

**Fig. 4:**
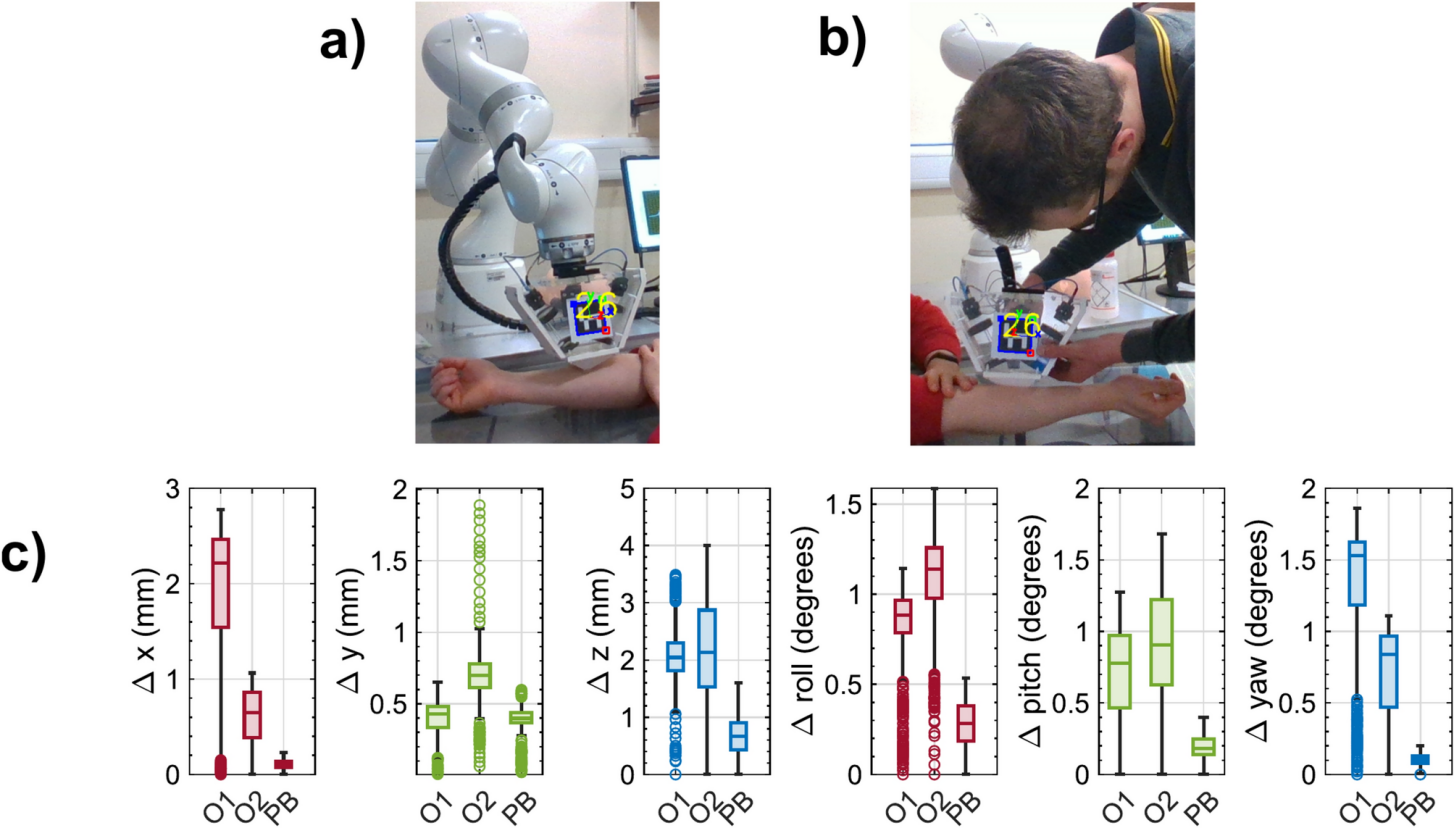
Comparison between the handheld system and the PicoBot for the stability during skin scanning using marker and camera (**a**) Pose estimation of the probe when attached to the robot (**b**) Pose estimation of the probe when handled manually (**c**) Variations of the estimated pose of the fiducial marker (ArUco tag) attached to the probe and compared between the manual Operator 1 (O1), manual Operator 2 (O2), and the PicoBot (PB) for three measurements of the same location. From left to right, it shows the variation of the position in x, y, and z followed by the orientation in roll, pitch, and yaw.

With the repeatability of the PicoBot system proved to be in a satisfactory range, we next show how the robot performs compared to the handheld system. Specifically, we compare the stability of the probe during the measurements using the visual stability check and then stability within the acquired data itself. Externally, a fiducial marker (ArUco tag) is attached to the probe as shown in Fig. 4a and b, and the pose of this tag is estimated from a camera fixed at a distance for 30 secs. Three repeated measurements are performed on the same volunteer by two manual operators and the PicoBot. The camera tracks the movement, by recording position and orientation of the marker when the probe is used in hand-held mode as well as with PicoBot. The small variation from the PicoBot tracker may occur due to the small motions of the subject (which the robot is trying to maintain) and the pose estimation error from the camera, which is valid for all cases. Figure 4c shows that the pose error of the PicoBot is significantly smaller than that of both operators for all the position parameters. From these data, we calculate the relative stability of the PicoBot to each operator as the average of the relative percentage error reductions for all the six position/orientation parameter errors. The PicoBot is found to be 68% and 73% more stable in positioning the probe compared to the manual operator 1 and operator 2 respectively.

**Fig. 5:**
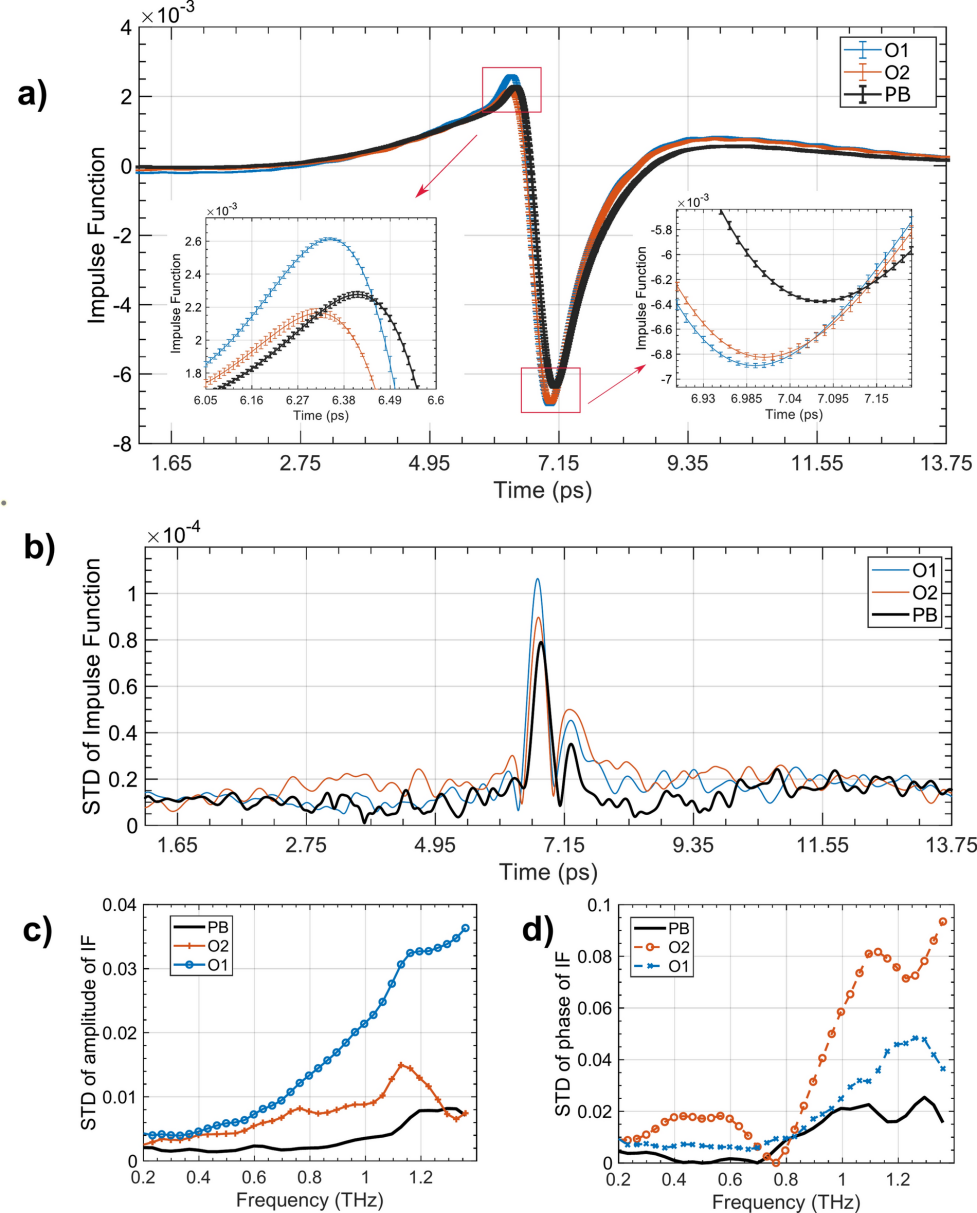
Comparison between the handheld system and the PicoBot for the stability during skin scanning using the THz response. (**a**) Averaged Impulse function and variation over the four repeated measurements of the volar forearm by O1, O2 and PB. (**b**) Standard deviations (STD) of the averaged impulse functions in (**a**). Standard deviations (STD) of the (**c**) amplitude and (**d**) phase of the averaged THz Impulse Functions (IF) over the frequency domain, with different operators (O1 and O2) and the PicoBot (PB).

To show the effects of the improved positional stability of the PicoBot on the THz measurement, Fig. 5a shows the mean processed THz pulses from the four repeated measurements of the volar forearm done by the two operators and the PicoBot. The zoomed-in insets show how the error bars (which are the standard deviation) are smaller overall for the PicoBot than for either operator. Similarly, Fig. 5b shows that the standard deviation of the THz pulse amplitude is lower for the PicoBot than either operator for most of the time window of the THz pulse. Figure 5c and d look at this in more detail by considering the amplitude and phase of IF in the frequency domain, and it is clear from these that the PicoBot measurements are much more consistent across the whole frequency range and are able to remove a lot of variation caused by having a human operator. For example, at 1 THz, the STD of the amplitude of IF measured by the PicoBot is less than four times smaller than by Operator 1. Therefore, from the comparative analysis, it can be concluded that the skin measurements are significantly more reliable, stable, and repeatable for the PicoBot system compared to the manual hand-held system.

### In vivo* human measurements*

Different areas on the human body namely the volar-forearm, fingers, cheeks, and the forehead are considered in this paper to demonstrate in vivo measurement possibilities with the PicoBot. Measurements of healthy skin and skin affected by eczema are also compared to demonstrate the diagnostic potential of the PicoBot. An assistant places markers around the intended ROI, before commanding the robot to move the safe distance off the skin. This is followed by initiating the force controller to make contact, and the THz controller is triggered to acquire data. The detailed protocol is given in the Measurement protocol section.

#### Healthy skin measurements

**Fig. 6:**
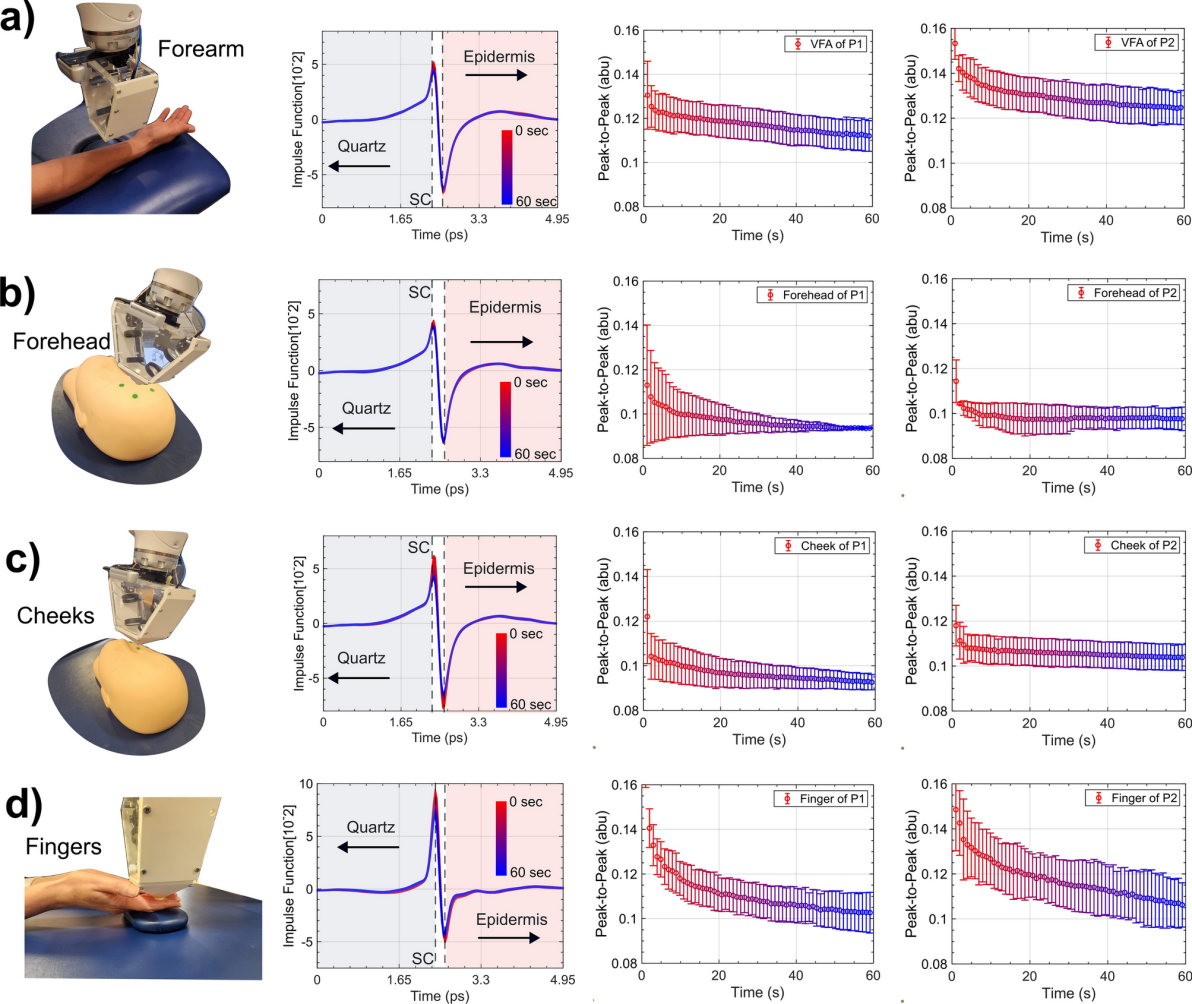
THz measurement set up and the results. From left to right we see the PicoBot measuring the ROI followed by THz pulses and occlusion curves recorded. Each of the two volunteers was measured three times in each of the four locations. Each measurement lasted for the 60 s. The setup and results are shown for measurements performed on the (**a**) volar forearm (**b**) forehead (**c**) cheek and (**d**) fingers of a human volunteer. The shaded error bars represent the standard deviations from the mean value for each set of three measurements. The human participant’s face has been replaced with a photo of the manikin being measured for anonymity.

Figure 6 shows that measurements with the PicoBot of a particular person and skin location are very consistent. In this Figure, each of the skin locations (forearm, forehead, cheek, and finger) were measured three times on two volunteers. As detailed in our previous work, there is a person-to-person^10^ and location dependent variation in the THz response of skin^11^, so some variation between subjects and locations is expected. The purpose of Fig. 6 is to highlight the repeatability of a measurement and in each of the subplots, we see that the error bars from the three measurements are small.

The volar forearm is the most convenient region for THz scanning as it is generally exposed or easily accessible. Furthermore, the volar forearm is typically smooth with little hairs allowing consistent contact with the THz probe head. Thus, many studies related to THz-based skin hydration are commonly performed on the volar forearm^10,36–38^. Figure 6a shows the THz response for the volar forearm surface when measured using PicoBot. The response of the THz pulses going through the quartz window of the probe and interacting with the skin is shown as an impulse function over 60 s. The peak-to-peak difference of the impulse function projected over the scanning period is termed as a peak-to-peak (P2P) curve or an occlusion curve. Figure 6b and c show the measurements on the forehead and cheeks of a human face. The human face is included in this study as it is the most common area for skin diseases and cancer, due to its exposure to direct sunlight. This is also the most challenging area to be scanned with a hand-held system due to the high curvatures observed in facial geometry. It is difficult to keep the hand-held system steady even for a minute in such areas. The THz measurement results for these challenging locations are shown in Fig. 6b and c respectively. The direct comparison of the occlusion curves between the two areas indicates a difference in hydration levels for each areas. These values can be used to compare skin conditions in the actual environment. A larger range of values in the occlusion curve indicates that the skin has to accumulate more hydration before becoming saturated, i.e., the skin is drier. On the contrary, a flatter occlusion curve indicates that the skin under examination is initially closer to being saturated and thus has a higher water hydration level initially. Figure 6d shows the repeated measurements on the finger site of the two volunteers and small error bars represent the consistent (stable) measurements over the time. The fingers are found to be the tricky areas to be scanned using a portable scanner in the handheld modality due to limited area or space leading to unstable probe positioning and they present an ideal case for highlighting the benefits of robot scanning.

#### Dry skin trials

**Fig. 7:**
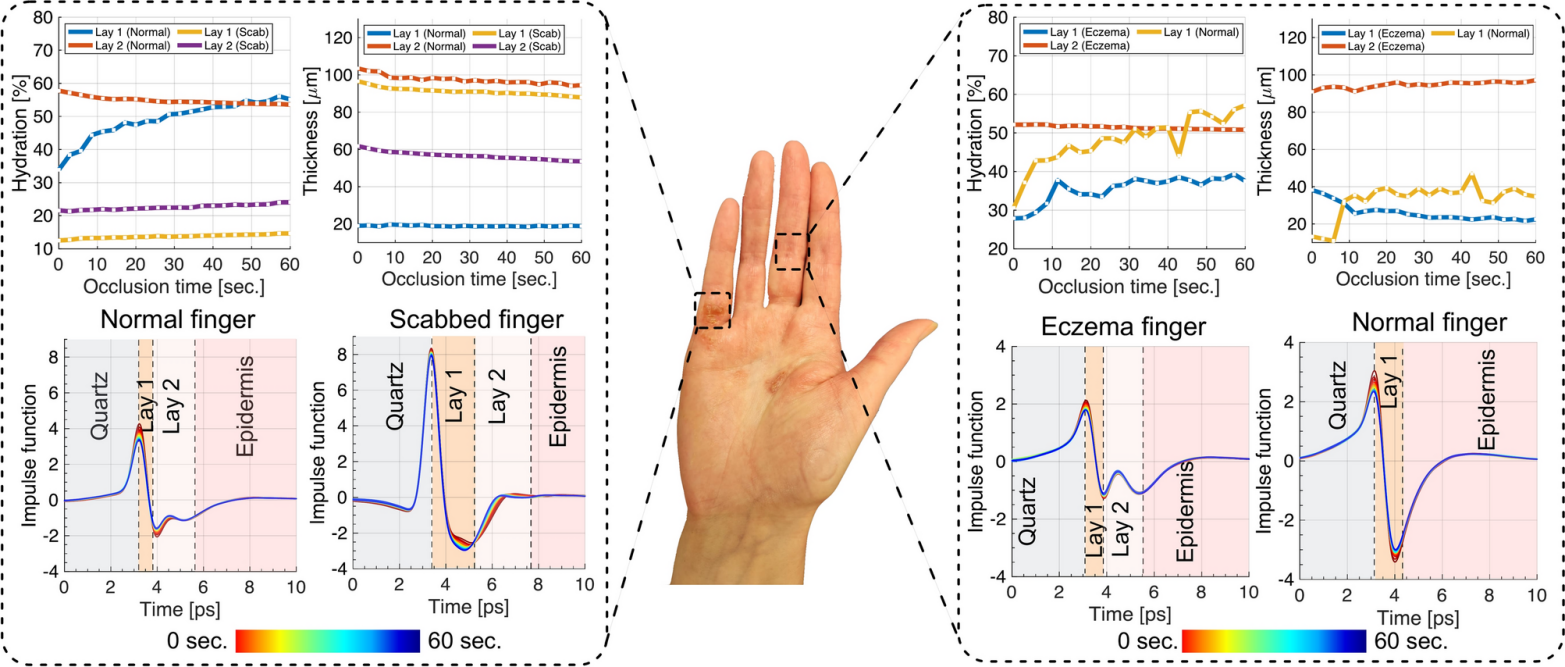
THz measurements for the fingers of a volunteer with the dry skin condition. Experimental measurements on the fingers of a volunteer suffering from eczema on one hand, results are compared with the same healthy regions on the other hand of the volunteer.

In this case study, a volunteer with a dry skin scab on the lower part of the little finger on the right hand and eczema at the medium part of the middle finger is inspected using the PicoBot, with results shown in Fig. 7. The healthy left hand was also measured for comparison. A four-layer model, as mentioned in supplementary section, was used to determine skin hydration and thickness. The four layers in sequence are: quartz window, dry skin, stratum corneum (SC), and epidermis. Although the skin has additional deeper layers, THz light can only penetrate down to the epidermis due to its high absorption coefficient, preventing deeper penetration. The dry skin on top of the SC is seen in eczema cases. As detailed in the analysis of the little finger in Fig. 7, the first layer (Lay 1) representing the scabbed area had the lowest hydration content at around $10\%$, while the same region of the healthy finger showed an increase from around $30\%$ to $60\%$ during a $60$ s scanning period, attributed to the occlusion effect. Additionally, the second layer (Lay 2), corresponding to the SC, exhibited an approximately $40\%$ hydration difference between the healthy and scabbed fingers. The technique also measured the scab thickness to be extending to nearly $100\, \upmu \text{m}$, compared to the healthy first layer at $20\, \upmu \text{m}$. The THz waveforms for both cases are presented in Fig. 7, showing a prominent peak in the Quartz-Lay1 interface for the scabbed finger, evidence of dry or dead skin. This peak was smaller for healthy cases, allowing for qualitative and quantitative dry skin evaluation. In another example, eczema on the middle area of the left middle finger was compared to the healthy finger on the opposite hand. Only three layers were visible in the healthy finger as no dry skin layer was present on top of the SC. The results revealed a $20\%$ higher water content in the healthy finger compared to the eczema finger. In short, THz sensing using the PicoBot can provide valuable insights that could be used to quantitatively evaluate the thickness and hydration of skin conditions.

## Discussions

This paper addresses the following questions: Can we measure human skin with THz light using a robotic system? How robust is the robot sampling system when compared with the hand-held probe? What level of autonomy can be achieved within the robotic system? Considering these points, the major contributions of the paper are as follows:

1. Development of a level $2$ to 3 autonomous robotic system to acquire THz measurements on the different parts of the body, modules of which can be initialized/interrupted/replayed by the operator following visual inspection and live feedback.
2. Implementation of the detection algorithm for segmenting and providing the robot with a target pose to scan.
3. Implementation of the cartesian impedance controller for safe physical human–robot contact, and a setpoint based force controller to apply a desired (reconfigurable) amount the force through a THz probe.
4. Safe, reliable and stable THz measurements of skin, even in hard to reach locations, with greater stability than the hand-held probe.

The vision for our PicoBot is that it will be able to conduct measurements of patients in a short time and could be used by GPs or even beauticians to evaluate skin conditions and determine the best treatment or beauty product. Typically, a single measurement would take 2–3 min to scan a single region starting from marker application, detection, and recording measurement data with the PicoBot. For a manual system, extended use of the heavy probe would lead to worsening positional accuracy and measurement quality. Our bench-top tests conducted demonstrate the preclinical feasibility of the concept along with the robustness and advantages of using the PicoBot.

Our marker-based technique for ROI segmentation is robust and fast without using any additional or pre-interventional images, and may be applied with limited technical knowledge. Markers may be applied on any region of the body, including the facial regions such as the cheeks, and forehead. Some regions, such as the nose, are significantly more challenging, as the small size and high curvature limit the visibility and placement of the markers. For these, we will focus our future works based on vision-based methods leveraging on Skinned Multi-Person Linear Model (SMPL) based body mesh for patients to facilitate fully marker less selection of ROI anywhere on the body. In the future, the PicoBot has the potential to autonomously scan the full body region without the intervention of an expert, leading to level 4 of robotic autonomy. The segmentation of the ROI for those with skin conditions is planned to be based on the abnormal skin regions rather than the markers.

A closed-loop setpoint-based force controller is vital for contact type measurements. Firstly, it is very difficult to hold the probe steadily in one hand for 60 s per measurement and then for multiple measurements. Secondly, it is important to monitor real-time contact force with the human body to ensure safety, to compensate for motion and force in the vertical direction while breathing, and to keep the desired contact pressure. Even though the manipulator has joint torque sensors, which are being used to compute the end-effector wrench, assuming a good load calibration, this can be further improved by using an external high-precision force torque sensor. The accurate force control system in the PicoBot scanner opens the possibility of extending these studies to a broader range of patients with the minimum discomfort for the participants when assessing diseased skin.

The extendibility of the current PicoBot setup is to acquire image measurements over the region of interest. It is not possible to slide the THz probe like an ultrasound probe as it might cause discomfort on the dry skin. Therefore, we are working on approaches for acquiring in vivo skin imaging using PicoBot. Recently published work by Qi et al.^39^ has used a quantum cascade laser (QCL) imaging approach to obtain high-speed in vivo THz images of skin pathologies. Although our approach is currently much slower than this, our measurements have been conducted at room temperature and have even been done in a hospital setting^40^**.** This contrasts to the QCL measurements which were done at 50 K in a laboratory environment (patients went to the university for the measurements). Now that we have shown how a robotic arm can be used to bring the THz probe into contact with the skin reliably and repeatably for point measurements, we will next incorporate hyperspectral imaging capabilities into the PicoBot. This could be done by using raster scanning of translation stages, or by using compressed sensing approaches as detailed in our previous work^41^**,** depending on what trade-off regarding acquisition speed versus spatial and spectral resolution is required. We envisage achieving five frames per second for suitable resolution hyperspectral images when we incorporate our compressed sensing approach with the robotic arm. These will be integrated into future PicoBot versions.

The PicoBot successfully positioned the THz scanner on a region of eczema on the little and middle finger of a volunteer—these are challenging areas to access through the handheld approach. However, using the PicoBot we were able to apply numerical algorithms^10^ to evaluate the accumulation of water in such affected areas. Future studies are planned to collect data from skin cancer patients and more subjects with dry skin conditions to further develop these applications. Our ultimate aim is to use the PicoBot to benefit patients for example by determining tumor margins without incision or biopsy, or being able to monitor skin conditions and generate tailored treatment plans. In this way, we envisage the PicoBot will reduce patient waiting times and improve patient outcomes.

## Methods

We conducted all in vivo measurements with human participants in our THz Laboratory at Warwick University. Ethical approval for the study was granted by the Biomedical and Scientific Research Ethics Committee (BSREC), Application number BSREC 26/23-24. Appropriate informed consent was taken from each human volunteer/participant before measurements and for using their pictures for this paper. All research was performed in accordance with the relevant guidelines and regulations, including in accordance with the Declaration of Helsinki. The approved protocol also included a short survey as it is important to keep a note of the patient’s recent activities which may affect the hydration level of the skin, such as, swimming, exercise, or bathing.

### THz probe

To record data in the THz experiments we use a TeraSmart THz-TDS spectrometer (Menlo Systems, Germany). A photoconductive antenna (PCA) made of $FE:InGaAs$ biased at $100\, \text{V}$ is excited by a femtosecond pulsed laser with center wavelength at $1550\, \text{nm}$ and temporal duration of less than $100\, \text{fs}$. This excitation generates electromagnetic transient with a temporal duration of less than $2\, \text{ps}$ having a spectral content between $0.1$ THz up to $5$ THz. This transient of radiation is referred to as a THz pulse. For the detection part, a non-biased PCA is connected to a trans-impedance amplifier and the signal detected is sent to a DAQ card connected to a computer. The femtosecond pulse is guided to the PCA using a couple of $2.5\, \text{m}$ optical fibers, providing us with the flexibility to position our THz system on the robotic manipulator. The THz scanner architecture is designed in an oblique reflection configuration with an incident angle of ${30}^{\circ }$ as illustrated in Fig. 1a. The tip of the scanner (probe) has a transparent $10\, \text{mm}$ diameter quartz window placed exactly at the focal point of the two THz lenses to help with the alignment of the scanner and to flatten the skin under examination improving the efficiency of the reflected THz light. In our PicoBot setup, we are displaying and recording THz pulses at the rate of 4 Hz, i.e., 4 pulses a second. As we measure for the 60 s, the total pulse count for PicoBot measurement is 240. We acquire our data using Python, implementing a node in ROS to trigger and collect recordings. The acquired data is further analyzed in MATLAB (MathWorks, USA).

### Robot manipulator control strategy

A 7-Degrees of Freedom (DoF) collaborative robot manipulator: LBR Med14 R820 (KUKA AG, Germany), is used in the proposed PicoBot setup. This manipulator is certified with its specific safety and quality standards for its integration into medical products. The manipulator is adaptable to meet medical requirements ranging from diagnostics to treatment and surgical procedures. The payload capacity of the Med14 version is $14\, \text{kg}$ with a reach of $820\, \text{mm}$ and repeatability of around $\pm 0.15\, \text{mm}$. We communicate with the robot manipulator through external ROS computer using Fast Robot Interface (FRI) monitoring the state of the robot such as joint position, joint commanded torques, joint external torques, and commanding the robot with joint position, velocity, and torques^42,43^*.* This is also integrated with different hardware and software modules such as depth camera, THz system, and other computational nodes. We modified the robot Unified Robot Description Format (URDF) files to include THz probe and used it to build a dynamic re-configurable Cartesian Impedance controller^44,45^*.*

For the Cartesian impedance controller implementation, the dynamic equation of motion of the controlled robot manipulator with gravity compensation can be written, in the joint space $q\in {R}^{n}$ as^46^,

1 $$M\left(q\right)\ddot{q}+C\left(q,\dot{q}\right)={\uptau }_{c}+{\uptau }_{ext}$$

where $,$ $n$ are the number of Degrees of Freedom (DoF) of the manipulator, $M\left(q\right)\in {R}^{n\times n}$ is the generalized inertial matrix, $C\left(q,\dot{q}\right)\in {R}^{n\times n}$ represents the Coriolis and centripetal forces, ${\uptau }_{c}\in {R}^{n}$ are the input torques, and ${\uptau }_{ext}\in {R}^{n}$ are the external torques. The input torques in the Cartesian impedance controller are the superposition of the 3 joint torques defined as,

2 $${\text{T}}_{c}={\uptau }_{ci}+{\uptau }_{ns}+{\uptau }_{ef},$$

where,

3 $$\uptau _{{ci}} = J^{T} \left( q \right)\left[ { - K^{{ci}} \Delta X - D^{{ci}} J\left( q \right)\left( {\dot{q}} \right)} \right]$$

4 $${\text{T}}_{{ns}} = \left( {I_{n} - J^{T} \left( q \right)\left( {J^{T} \left( q \right)} \right)^{\dag } } \right)\left[ { - K^{{ns}} \left( {q - q^{d} } \right) - D^{{ns}} \dot{q}} \right]$$

5 $${\text{T}}_{ef} = J^{T} \left( q \right)F_{ext} .$$

Here, ${J}^{T}\left(q\right)\in {R}^{m\times n}$ is the Jacobian matrix of the manipulator, ${K}^{ci}\in {R}^{m\times m}$ and ${D}^{ci}\in {R}^{m\times m}$ are the Cartesian stiffness and damping matrices of the virtual spring at the tool-tip of the manipulator respectively, whereas ${K}^{ns}\in {R}^{n\times n}$ and ${D}^{ns}\in {R}^{n\times n}$ are the virtual joint stiffness and damping matrices to achieve joint impedance behavior for the null space of the manipulator without affecting the Cartesian motion of the tool-tip frame.$\Delta X$ is the Cartesian pose error, that is the error between the position and orientation of the tool-tip frame with respect to the desired set-point pose. $\left( {J^{T} \left( q \right)} \right)^{\dag }$ is the Moore–Penrose pseudo inverse of the Jacobian matrix, and ${F}_{ext}$ is the desired external wrench in the tool-tip frame of the manipulator.

### Software architecture

The software architecture developed and integrated for the PicoBot is briefly described in this section. This incorporates various modules with corresponding functionalities and interdependency with each other to aid autonomy and robustness of the whole system. Complete architecture can be categorized into two major portions. One is for the Autonomous Robot Motion plan and Force (ARMoFo) application system, and another is for the autonomous THz data acquisition (ATDA) system. The architecture is built over a ROS platform exploiting low-level controls with the robot and building high-level nodes for system functionalities which are briefed in the following sections. Each node in both systems communicates over various ROS topics through publisher/subscriber and service/request channels. The ARMoFo system includes the following nodes. (i) A Graphical User Interface (GUI) node for easy interaction between the user and the PicoBot System. (ii) Cartesian impedance controller node to active impedance control mode with dynamic reconfiguring of stiffness parameters. (iii) Camera node for RGB-D images along with the eye-in-hand calibration parameters and PCL filters. (iv) Target detection and localization node, along with the supporting nodes for surface normal and corresponding pose computations, transformation of the data from the camera frame to the robot base frame. (v) Cartesian trajectory planner and execution node for the end-effector motion of the manipulator based upon the detected target. (vi) Jacobian, Wrench computation node with respect to the end-effector of the manipulator. (vii) Force controller node for the set-point based indirect force control for the contact between end-effector (tooltip) and the human skin surface.

Similarly, the ATDA system, which also communicates with ARMoFo includes, (i) wrench subscription node at $4Hz$ for triggering the data acquisition. (ii) THz pulse starting/stopping and recording node to save or visualize the measurement data online. (iii) THz measurement data post-processing to analyze useful information.

### Target detection and localization

**Fig. 8:**
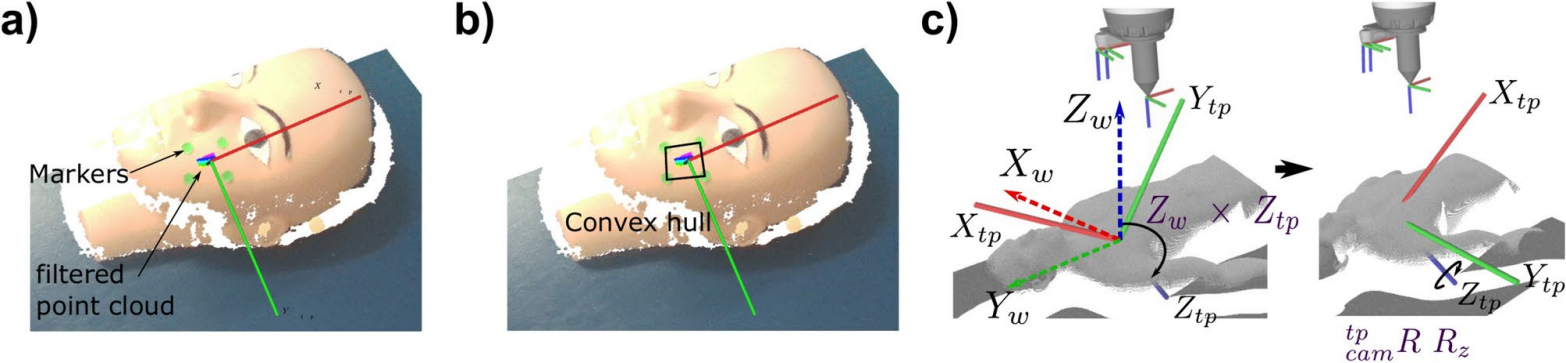
Marker detection and pose estimation (**a**) 4 green-colored marker detection on the cheeks region using Yolo-V5 detection for point cloud segmentation and surface normal estimation, (**b**) Convex hull generated from the markers and defining the center point to estimate the pose at the point, (**c**) Pose estimation and aligning target orientation with end-effector tip coordinate frame.

To perform THz scanning on the ROI, the THz probe is required to be placed in contact with the skin and oriented perpendicular to the skin surface. To perform this task autonomously, the target detection and localization algorithms are developed based on marker-based detection and pose estimation using the obtained point cloud data in real-time from the vision module. Four green circular markers are attached to the ROI to segment out the point cloud from the whole image. The detection algorithm for these markers is developed using You Only Look Once Version $5$ (YOLOV5) due to its high accuracy and real-time detection performance^47^. YOLOV5 is a single-stage object detection algorithm that uses Deep Convolutional Neural Network (dCNN) that treats object detection as a regression problem, and outputs the bounding box coordinates for each object detected in a given image. A custom data set of $400$ images, containing green circular markers on the skin with different skin tones, is used to the train the model. These data images are collected with different lightning conditions and appropriate image augmentation through scaling, rotation, flipping, exposure, brightness and hue changes are added to improve training of the network. The detected marker bounding boxes are used to extract the $2\text{D}$ marker centers $\left({x}_{i}, {y}_{i}\right).$ A convex hull is fitted in order to extract the scan region, and the THz probe is planned to be placed at the hull center (${x}_{c},{y}_{c}$), as shown in Fig. 8b. To convert the $2\text{D}$ pixel coordinates to $3\text{D}$ coordinates in the camera frame, the intrinsic and extrinsic properties of the RGB-D camera are used.

To localize the target with respect to the robot base frame, eye-in-hand calibration is performed using the Moveit calibration package^48^ as shown in Fig. S1. It provides plugins and a graphical user interface using an ArUco based target mesh, RGB images and camera intrinsic parameters to compute the relative coordinate frame. For better calibration results, multiple samples with different poses and orientation about every axes were taken as suggested in^49^. The eye-in-hand was verified using a pointy tool and a grid sheet, and manual adjustments were made to improve accuracy.

For the pose computation, first the dense point cloud is filtered out for the area of interest segmented by the detection algorithm using the crop box filter and voxel-grid filter^50^, as shown in Fig. 8a. The filtered point cloud is used to the estimate the surface normal at each point by performing eigen decomposition of the k-neighborhood points. The eigen vector corresponding to the smallest eigen value represents the surface normal at that point. Now, there might be an infinite number of possibilities to generate $3$ orthonormal axes (including normal) to define a pose. To estimate the pose systematically first the cross-product is performed between the world ${Z}_{w}-$ axis, which is $\left[0, 0, 1\right]$ with the normal at the point (${Z}_{tp}$), see Fig. 8c. The resultant and normalized cross-product vector is defined as ${X}_{tp}-$ axis. Second, the resultant cross-product of the ${Z}_{tp}$ to ${X}_{tp}$ gives the ${Y}_{tp}-$ axis. Each vector of ${X}_{tp},{Y}_{tp}$ and ${Z}_{tp}$ is reformatted as a column vector of a $3\times 3$ matrix and represents a rotation matrix with respect to the camera frame. The obtained orientation is aligned with the tool frame using ${}_{tp}{}^{cam}R = {}_{t{p}{\prime}}{}^{cam}R{R}_{z},$ where ${R}_{z}$ is the rotation operator about $Z-$ axis represented as a rotation matrix. This is performed for unnecessary rotation of the tool in the motion planning. Finally the target pose with respect to the camera optical frame can be represented as,

6 $${}_{tp}^{cam} T = \left[ {\begin{array}{*{20}c} {{}_{tp}^{cam} R} & {^{cam} p} \\ 0 & 1 \\ \end{array} } \right]$$

where, $^{cam} p$ represents the position of the target point with respect to the camera frame. These target points are actually the set of poses on each point based upon down-sampled points. For the robot to perform a measurement on the spot, either any point can be selected and sent to the robot or the pose corresponding to the central point of the convex hull can be considered. The latter method is more robust and is able to tackle the random noise of the depth cloud of the camera. To perform this, a weighted average of all the poses in the ROI is computed by the following equation,

7 $$\text{P} =\frac{{\sum }_{i=0}^{n}{w}_{i}{p}_{i}}{{\sum }_{j}{w}_{i}{p}_{i}},$$

8 $$w_{i} = \left\{ {\begin{array}{*{20}l} {c.exp\left\{ {\frac{1}{{4\left( {d_{i}^{2} - d_{{max}}^{2} } \right)}}} \right\},} \hfill & {d_{i} < d_{{max}} } \hfill \\ {0,} \hfill & {d_{{max}} \le d_{i} } \hfill \\ \end{array} } \right.$$

where ${w}_{i}$ are the weights computed using Eq. (8) and ${d}_{i}$ are the Euclidean distances of the ${i}^{th}$ pose from the center, and ${d}_{max}$ is chosen to be 0.5. The weights are taken from a kernel often used in mathematical analysis to approximate non-smooth functions by smooth ones by convolution or mollification. The weights attribute higher priorities to the surface normal closer to the center of the scan region. Finally, ${}_{tp}{}^{b}T$, which represents the single point central pose of the ROI with respect to the base of the manipulator can be computed from the forward kinematics of the robot and eye-in-hand calibration, is sent to the robot controller for motion planning and execution. The pose obtained with the proposed methodology lies over the skin surface of the target area to be scanned. To keep a safe distance from the human, the tool tip is commanded to move to a pose which is just above the surface by $\Delta Z$, with respect to the tool tip frame of reference, and can be computed as,

9 $${}_{tp}{}^{b}{T}_{safe}={}_{tp}{}^{b}T \left[\begin{array}{cc}{\varvec{I}}& {\left[\text{0,0},-\Delta Z\right]}^{T}\\ 0& 1\end{array}\right]$$

### Set-point based force control

Once the tooltip of the robot has been moved to the target pose with safe distance, and keeping the orientation normal to the skin surface, the next step is to make contact with the skin surface with a desired amount of force and trigger the THz system to initialize measurements. The manufacturer of medical grade KUKA LBR provides a low-level controller with automatic gravity compensation of the manipulator dynamics. A properly calibrated gravity-compensated model should ideally reflect zero external torques in each joint. Adding an end-effector or tool to the manipulator requires the recalibration of the robot dynamics, which involves identifying the inertial parameters of the attached tool. To perform this calibration, KUKA controller provides the interface to perform, what they called, load data determination.

**Fig. 9:**
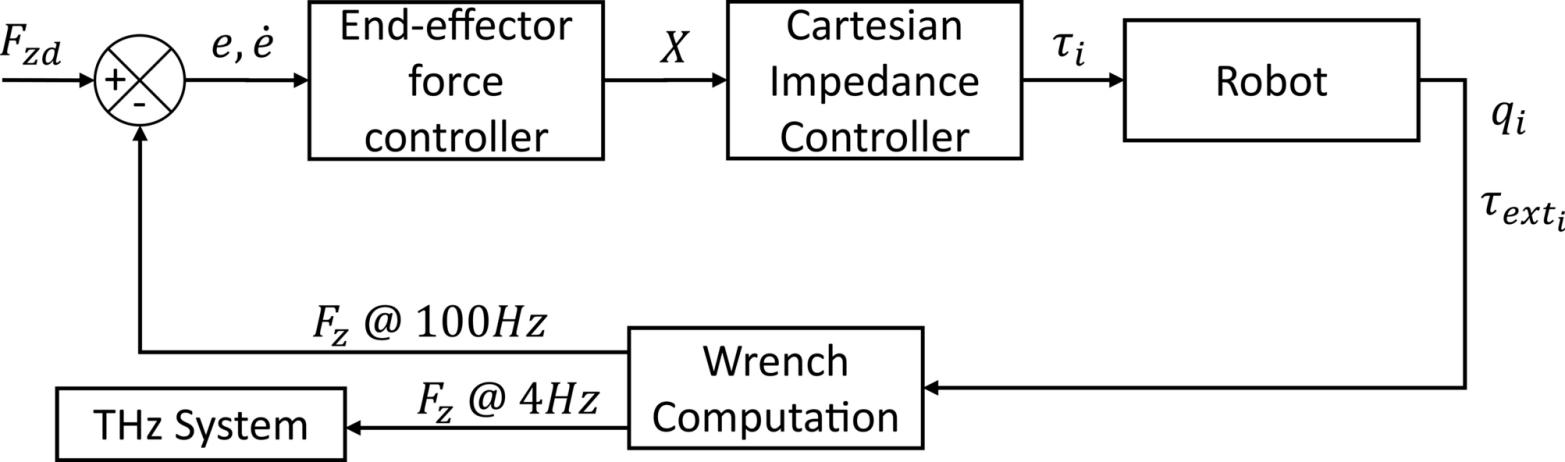
External sensor less set-point based force controller. A closed loop end-effector force controller acting in the direction of the Z-axis of the end-effector tip, through which the wrench computation node is sending sensed and computed wrench messages at different rates.

KUKA LBR Med has inbuilt torque sensor at each joint, which also provides the external torque values at each joint when the manipulator is in contact. In the quasi-static condition, the measured external joint torques (${\uptau }_{ext}\in {R}^{7}$) can be used to compute the external wrench $\left({F}_{ext}\in {R}^{6}\right)$ at the tip of the manipulator end-effector using the expression,

10 $$F_{ext} = \left[ {J^{T} } \right]^{\dag } \tau_{ext} ,$$

where $\left[ {J^{T} } \right]^{\dag }$ is the pseudo inverse of the transpose of the Jacobian. Here, the pseudo inverse is required because the $J\in {R}^{7\times 6}$. This helps in cutting the cost of the PicoBot system by discarding the use of any commercial and expensive force-torque sensor. The wrench is computed in the base coordinate frame of the manipulator and are then transformed to the tool tip coordinate frame. It is then published on the two topics through the ROS system, one at $100 \text{Hz}$: required for the force controller, and the other at $4 \text{Hz}$: required for the THz system, as shown in Fig. 9.

A set-point based indirect force control strategy is applied in this work benefiting from the Cartesian impedance behavior of the end-effector of the manipulator. The computed wrench using Eq. (10) at $100 \text{Hz}$ is used in the closed loop force controller, as shown in Fig. 9. The desired set-point ($X$), which is fundamentally a very small displacement of desired position and orientation from the current position of the tool tip, is calculated. The desired set-point is fed to the Cartesian impedance controller to compute the required joint torques to move the robot as in Eq. (5). A set-point controller is found to be very stable especially in the vertical motions of the contact surface for example while breathing, which is important for $\text{in vivo}$ measurements.

## Supplementary Information

Supplementary Information 1.

Supplementary Information 2.

Supplementary Video 1.

Supplementary Information 3.

## Data Availability

Data is provided within the manuscript or supplementary information files.
