# Supplementary material for: Towards autonomous robotic THz-based in vivo skin sensing: the PicoBot
Source: Sci Rep. 2025 Feb 7;15:4568. doi: 10.1038/s41598-025-88718-6 (PMC11803113; doi:10.1038/s41598-025-88718-6)
Supplement: Supplementary file 1 — Supplementary Information 1. [file 41598_2025_88718_MOESM1_ESM.docx]

**Towards Autonomous Robotic THz-Based *In Vivo* Skin Sensing: The PicoBot**

Anubhav Dogra^1,*^, Dominic Jones^2^, Arturo Ignacio Hernandez Serrano^1^, Shruti Chakraborty^1,†^, Jacob Joshua Young^1^, Benjamin George Page^1^, Joseph Hardwicke^3,4^, Pietro Valdastri^2,*^, Emma Pickwell-MacPherson^1*^

^1^Department of Physics, University of Warwick, United Kingdom,

^2^STORM Lab, University of Leeds, United Kingdom,

^3^Warwick Medical School, University of Warwick, United Kingdom,

^4^Institute of Applied and Translational Technologies in Surgery, University Hospitals Coventry and Warwickshire NHS Trust, Coventry, United Kingdom

email: [anubhav.dogra@warwick.ac.uk](mailto:anubhav.dogra@warwick.ac.uk), [p.valdastri@leeds.ac.uk](mailto:p.valdastri@leeds.ac.uk), [e.macpherson@warwick.ac.uk](mailto:e.macpherson@warwick.ac.uk)

**Supplementary Materials**

- Movie S1
- Fig. S1 Transformation frames of the robot system.
- Supplementary Materials and Methods

**Movie S1:** The movie shows the individual recordings for different working processes of the PicoBot system, including marker-based segmentation and localization, force controller, and GUI demonstration.

**Figure S1. Transformation frames of the robot system,** depicting the camera to base calibration in eye in hand setup and pose at the target region with respect to the base of the robot.

**Supplementary Methods**

**Vision Module**

The target location information is needed to command the robot to position the tool tip on the target point. The target point needs to be conveyed to the robot controller with respect to the robot base frame or the world frame defined in the system. To get the precise transformation information between the target point and the robot base, a precise vision system is required. To achieve that, a compact 3D scanner from *RealSense* with RGBD information is selected for the setup. The compact design of the 3D camera means it can be mounted directly onto the robot and is in eye-in-hand configuration. The eye-in-hand calibration information is shown in Fig. S1, where ${}_{b}^{ee}T$ represents the transformation of end-effector frame with respect to the robot base frame, ${}_{ee}^{cam}T$ represents the transformation of the camera optical frame with respect to the robot end-effector frame (eye-in-hand calibration), ${}_{ee}^{tip}T$represents the transformation of tool tip frame with respect to the robot end-effector frame, and ${}_{cam}^{tp}T$ represents the transformation of target point frame with respect to the camera optical frame. Using all these transformations, the pose of the target point can be represented in the base frame as ${}_{b}^{tp}T$ as described in Eq. S1.

${}_{tp}^{b}{T=}{}_{ee}^{b}{T {}_{cam}^{ee}T {}_{tp}^{cam}T}$ (S1)

To align the tool tip pose with the target point pose, ${}_{b}^{tip}T$ has to be equal to ${}_{b}^{tp}T.$

**THz Data Processing**

The THz light is highly absorbed by hydrated materials due to the high absorption coefficient of water ($200 \text{c}\text{m}^{\text{-1}}$ at $1$ THz), therefore with this technique, the skin depth assessment is limited to a few hundred microns. The topmost layer of skin, the stratum corneum (SC), which is the first protective barrier of the body against external agents (viruses pathogens, among others) extends up to $200 \mu m$ in some parts of the body, and is therefore accessible for sensing using THz light. The reflected total field is compared with a mathematical model to retrieve the hydration levels and thickness of skin layers. This mathematical model is based on the electromagnetic theory of multi-layer systems, in particular, the four-layer model. To build up this model, it's worth starting from the three layers equation (*47*),

$r_{123,s}=\frac{r_{12,s}+r_{23,s\text{exp}}\left( -2i\beta_{2} \right)}{1+r_{12,s}r_{23,s\text{exp}}\left( -2i\beta_{2} \right)},$ (S2)

where, $r_{123,s}$ is the complex reflection coefficient from the layers 1, 2 and epidermis, as shown in Fig. 1**,** for $s$ polarization; $r_{ij,s}$ stands for the Fresnel coefficient of reflection in the interface $ij$ for $s$ polarization; $\beta_{2}=k_{0}dn_{2\text{cos}}\theta_{2}$, where $k_{0}$is the propagation constant in vacuum, $n_{2}$ is the refractive index of layer 2, $d_{2}$ is thickness and $\theta_{2}$ is the incident angle. Then, the four layers model is obtained using Eq. (S2)

$r_{0123,s}=\frac{r_{01,s}+r_{123,s\text{exp}}\left( -2i\beta_{1} \right)}{1+r_{01,s}r_{123,s\text{exp}}\left( -2i\beta_{1} \right)},$ (S3)

where, $r_{123,s}$ is given by Eq. (S2), $\beta_{1}=k_{0}d_{1}n_{1\text{cos}}\theta_{1}$ and $d_{1},n_{1}$ and $\theta_{1}$ are the thickness, refractive index and incident angle into layer 1, respectively. Using a non-linear least squares routine, the theoretical model is fitted with the experimental electric field optimizing the refractive index and thickness of layers 1 and 2. In biological samples, the associated refractive index is directly related with its water levels, hence, this methodology permits the assessment of two crucial characteristics of skin in a non-invasive fashion.
