# Supplementary figures and images for: Towards autonomous robotic THz-based in vivo skin sensing: the PicoBot
Source: Sci Rep. 2025 Feb 7;15:4568. doi: 10.1038/s41598-025-88718-6 (PMC11803113; doi:10.1038/s41598-025-88718-6)

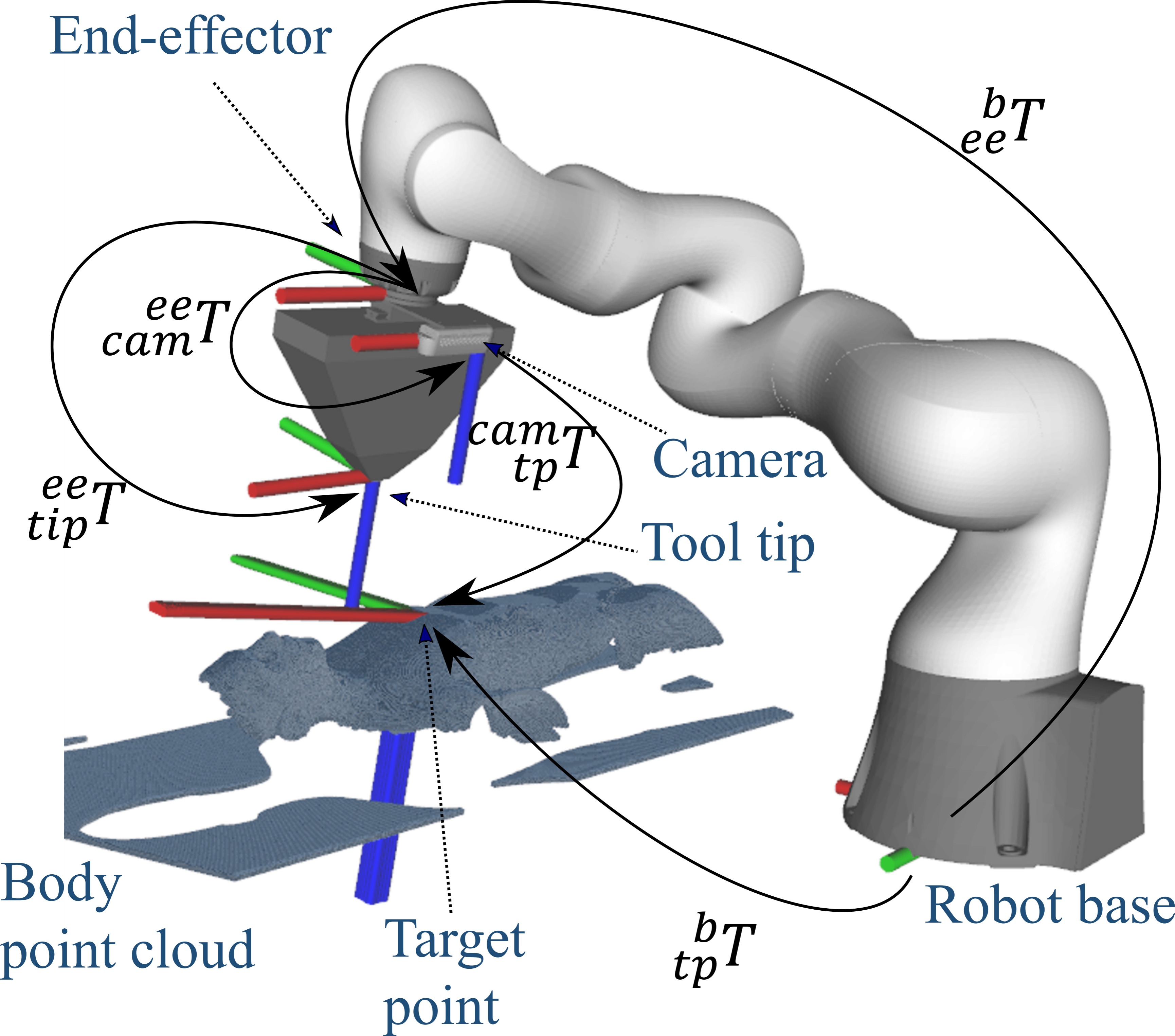

Supplement: Supplementary file 2 — Supplementary Information 2. [file 41598_2025_88718_MOESM2_ESM.pdf]
